# Evaluation of a newly designed deep learning-based algorithm for automated assessment of scapholunate distance in wrist radiography as a surrogate parameter for scapholunate ligament rupture and the correlation with arthroscopy

**DOI:** 10.1007/s11547-023-01720-8

**Published:** 2023-09-20

**Authors:** Gabriel Keller, Katarzyna Rachunek, Fabian Springer, Mathias Kraus

**Affiliations:** 1 Department of Diagnostic and Interventional Radiology, University Hospital Tübingen, Eberhard Karls University Tübingen, Hoppe-Seyler-Str. 3, 72076 Tübingen, Germany; 2 Department of Diagnostic Radiology, BG Trauma Center Tübingen, Eberhard Karls University Tübingen, Tübingen, Germany; 3 Department of Hand, Plastic, Reconstructive and Burn Surgery, BG Trauma Center Tübingen, Eberhard Karls University of Tübingen, 72076 Tübingen, Germany; 4 Institute of Information Systems, FAU Erlangen-Nuremberg, Nuremberg, Germany

**Keywords:** DL, AI, Automated, Scapholunate, SLAC

## Abstract

**Purpose:**

Not diagnosed or mistreated scapholunate ligament (SL) tears represent a frequent cause of degenerative wrist arthritis. A newly developed deep learning (DL)-based automated assessment of the SL distance on radiographs may support clinicians in initial image interpretation.

**Materials and Methods:**

A pre-trained DL algorithm was specifically fine-tuned on static and dynamic dorsopalmar wrist radiography (training data set *n* = 201) for the automated assessment of the SL distance. Afterwards the DL algorithm was evaluated (evaluation data set *n* = 364 patients with *n* = 1604 radiographs) and correlated with results of an experienced human reader and with arthroscopic findings.

**Results:**

The evaluation data set comprised arthroscopically diagnosed SL insufficiency according to Geissler’s stages 0–4 (56.5%, 2.5%, 5.5%, 7.5%, 28.0%). Diagnostic accuracy of the DL algorithm on dorsopalmar radiography regarding SL integrity was close to that of the human reader (e.g. differentiation of Geissler’s stages ≤ 2 versus > 2 with a sensitivity of 74% and a specificity of 78% compared to 77% and 80%) with a correlation coefficient of 0.81 (*P* < 0.01).

**Conclusion:**

A DL algorithm like this might become a valuable tool supporting clinicians’ initial decision making on radiography regarding SL integrity and consequential triage for further patient management.

## Introduction

Artificial intelligence (AI) is an umbrella term for mathematical algorithms making intelligent decisions without human intervention [1]. Recently, deep learning (DL) algorithms became a matter of particular interest in medical research. Based on pre-trained neuronal networks and further specific training these are potentially able to make adequate decisions in new and unknown situations of the same character [2, 3]. DL algorithms are currently used in radiological research for image acquisition, for example, in order to minimize acquisition time for magnetic resonance imaging (MRI) [4–6]. Another field of interest is the automated image interpretation, for example in dynamic MRI or conventional radiography [7–10]. The aim of DL-based automated image interpretation is to reach a high sensitivity and specificity.

The scapholunate ligament (SL) is an important component of wrist stability. SL tears and consecutive insufficiency might lead to scapholunate advanced collapse (SLAC), which represents the most common cause of posttraumatic wrist degeneration [11]. To prevent the progression from SL tear to wrist degeneration early diagnosis and therapy is mandatory [12]. Image modality of choice for patients with suspected SL tears without the risks of an invasive method is MRI. Invasive methods such as CT arthrography or MRI arthrography has been reported superior to MRI [13] and arthroscopy remains the gold standard for the clarification of a suspected SL tear [14]. Plain radiography of the wrist is most suitable for initial triage of patients with suspected SL tears [15]. However, detecting potential SL tears on radiographic images can be challenging. Therefore, the development of a reliable automated assessment of SL integrity could be of significant clinical value in the early detection and furthermore the early treatment of SL tears. DL algorithms might provide this support for the interpreting radiologist or surgeon in this important initial decision making.

This manuscript makes use of recent advances in the field of DL to develop an algorithm for wrist radiography in order to detect SL dissociation as a surrogate parameter for SL tears and the evaluation of this algorithm in comparison to the radiological reports and arthroscopic findings of the same patients.

## Materials and methods

### Study design

The institutional review board approved the study design (review board Application Number 600/2021BO2). The study was conducted in accordance with the Declaration of Helsinki (as revised in 2013).

**Fig. 1:**
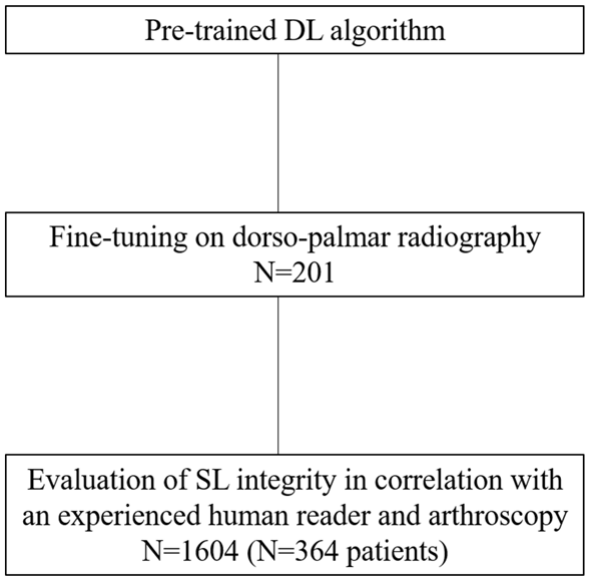
Flowchart of the process of development and evaluation of the newly DL algorithm for the assessment of SL integrity on dorsopalmar radiography. DL = deep learning, SL = scapholunate ligament

A pre-trained DL algorithm was specifically trained on wrist radiographs in dorsopalmar view (training data set *n* = 201; 50.7% right hand, 49.3% left hand, 31.8% ulnar abduction, 30.8% radial abduction, 37.3% neutral hand position). These were randomly selected out of the institutional picture archiving and communication system (PACS) in July 2021. There were no specific exclusion criteria (Fig. 1).

Thereafter the DL algorithm analyzed an evaluation data set (*n* = 364 patients with *n* = 1604 radiographs) of wrist radiographs of patients with clinically diagnosed wrist pain and suspected SL tear and corresponding radiographs of the contralateral unaffected hand. Inclusion criteria was the presence of a timely associated arthroscopic correlation within a maximal time interval of three months. There were again no specific exclusion criteria. The radiographs were out of the study period October of 2010 till December of 2017.

The results of the DL algorithm were correlated with results of an experienced human rater and the arthroscopic results.

### Image acquisition of the radiographs

All radiographs for both the training data set and the evaluation data set were extracted from the institutional PACS. Respected were all radiographs in dorsopalmar view, in neutral hand position as well as in radial abduction and in ulnar abduction. Most of the radiographs were manufactured on one of the three institutional X-ray scanners (PCR Eleva, Philips Medical Systems DMC, Hamburg, Germany) with dose relevant parameters of the standard wrist protocol set to 52 kV and 2.5 mAs. Clinic’s patients’ radiographs from external institutions acquired on different X-ray scanners from different vendors and with different study protocols, which had been transferred to the institutional PACS radiographs for diagnostic and therapeutic reasons were also included.

### Development of a DL algorithm for automated assessment of scapholunate distance

A DL algorithm was developed for automated assessment of scapholunate distance in wrist radiographs using the Mask RCNN Resnet 50 FPN model [16]. Mask RCNN is a state-of-the-art DL architecture that combines object detection and instance segmentation tasks. The model was pretrained on the Common Objects in Context (COCO) data set [17].

The pretrained model was then fine-tuned to segment the contours of the scaphoid and the lunate on the radiographs. For this purpose, the peripheral bone contour of scaphoid and lunate was given in the training data set, regardless of overlays of cortical and medullary bone or of different bones. The smallest distance between the two segmented bones was defined as the SL distance. For fine-tuning a data set of *n* = 201 wrist radiographs with varying degrees of SL dissociation was used. The model was trained for accurate segmentation of the scaphoid and lunate. To avoid overfitting and ensure robust performance, the average precision (AP) was tracked on a validation set of *n* = 80 wrist radiographs, and we evaluated this performance every 250 epochs. Our rationale behind this spacing was to prevent any premature conclusions based on isolated outcomes on the relatively small validation set for single epochs that might not correctly represent the model´s performance on an unseen test set. The base learning rate was set to 0.00–025, the weight decay to 0.0001, and the model was trained for 2000 optimization steps, after which no further improvements were observed on the validation set. A batch size of 2 was used to balance the trade-off between memory usage and computational efficiency. We implemented the DL algorithm using the Python package detectron2 v0.6 and used default preprocessing steps of this package. All training and inference code is publicly available at www.github.com/blinded-for-review.

### Evaluation of the SL integrity by the DL algorithm, a human reporter and arthroscopy

According to the training the DL algorithm segmented the scaphoid and the lunate and measured the smallest SL distance (mm) between the two bones. The human reporter (five years of experience in dedicated wrist radiography) measured the SL distance (mm) according to the recognized cortical contour of the scaphoid and the lunate. Since there is no international consensus for the interpretation of a normal SL distance, results were analyzed according to recent literature and most common practice with cut-offs at both ≤ 2 mm versus > 2 mm and at ≤ 3 mm versus > 3 mm [15, 18]. In addition, optimal thresholds were determined for the DL algorithm. We calculated the sensitivity and specificity for both the DL algorithm and the human reporter.

The arthroscopic SL integrity was analyzed based on surgical reports on the Geissler’s classification [19].

### Statistical analysis

Statistical analysis was performed using the software package scipy (version 1.10.0). The performance of the algorithm was evaluated and is presented by receiver operating characteristic (ROC) curve. Additionally, we determined the optimal threshold for the DL algorithm when predicting the SL integrity using Youden's J statistic [20]. We calculated the J statistic for a range of possible threshold values, and selected the value that maximized J. The resulting threshold was then used to predict the arthroscopic SL integrity as positive or negative based on the DL algorithm's SL distance measurement.

As a proxy measurement to filter out observations of misclassified scaphoid or lunate, we additionally analyzed the subgroups of radiographs that had a predicted SL distance below 5 mm and 7 mm, respectively.

We compared the DL algorithm's classifications to those of the human reporter and arthroscopy, which was considered the gold standard for diagnosing the SL integrity with the Spearman's rank correlation coefficient. The SL distances from the human reporter was compared to the SL distances from the DL algorithm using the Pearson's correlation coefficient.

## Results

### Study population

The evaluation data set comprised a male predominance (*n* = 267; 73.4%) compared to female patients (*n* = 97; 26.6%), mean age was 47.5 [± 12.8] years with a total range of 10–78 years. Out of the 1604 images in the evaluation data set, 791 are from the left hand, 813 from the right hand, 532 in radial abduction, 534 in ulnar abduction, and 538 in neutral hand position.

Arthroscopically results of SL integrity in the evaluation data set included all stages according to Geissler’s [19]: 56.5%, 2.5%, 5.5%, 7.5%, 28.0% (stage 0–stage 4). In three patients the Geissler’s stage of SL integrity was not mentioned in surgical reports.

### Evaluation of SL integrity by the human reporter

**Fig. 2:**
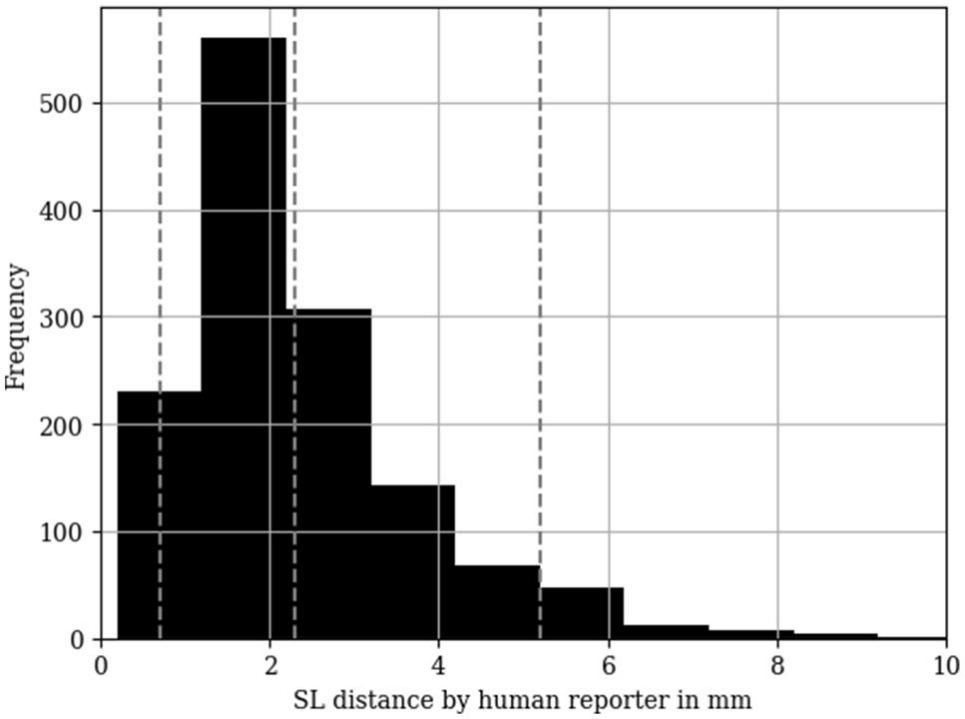
Histogram of SL distances measured by the human reporter in the study population (*n* = 364 patients; *n* = 1604 radiographs). Dashed lines mark the mean, the 5th and the 95th percentile. SL = scapholunate

The human reporter observed an average SL distance of 2.3 mm, with a range spanning from 0.2 mm to 10.2 mm. The 5th and 95th percentiles were 0.7 mm and 5.2 mm, respectively, as illustrated in Fig. 2. Of the reported measurements, 46% exhibited a SL distance greater than 2 mm, and 22% showed a SL distance exceeding 3 mm.

The spearman’s rank correlation coefficient between the measured SL distance of the human reporter and the Geissler’s classification was 0.58 (*P* < 0.01). Table 1 describes the sensitivity and specificity for predicting the arthroscopic SL integrity based on the human reports of the SL distance.

**Table 1:** Sensitivity and specificity of an experienced human reporter assessing the SL distance on dorso-palmar radiography as a surrogate parameter for the prediction of arthroscopic SL integrity

Geissler’s classification	Cut-off (mm)	Sensitivity	Specificity
0 versus > 0	≤ 2 versus > 2	0.85	0.54
0 versus > 0	≤ 3 versus > 3	0.74	0.86
≤ 1 versus > 1	≤ 2 versus > 2	0.86	0.53
≤ 1 versus > 1	≤ 3 versus > 3	0.75	0.84
≤ 2 versus > 2	≤ 2 versus > 2	0.88	0.50
≤ 2 versus > 2	≤ 3 versus > 3	0.77	0.80

Various cut-off values for the SL distance for the discrimination of different stages of Geissler’s classification [18] are reported

*SL* scapholunate

### Evaluation of SL integrity by the DL algorithm

**Fig. 3:**
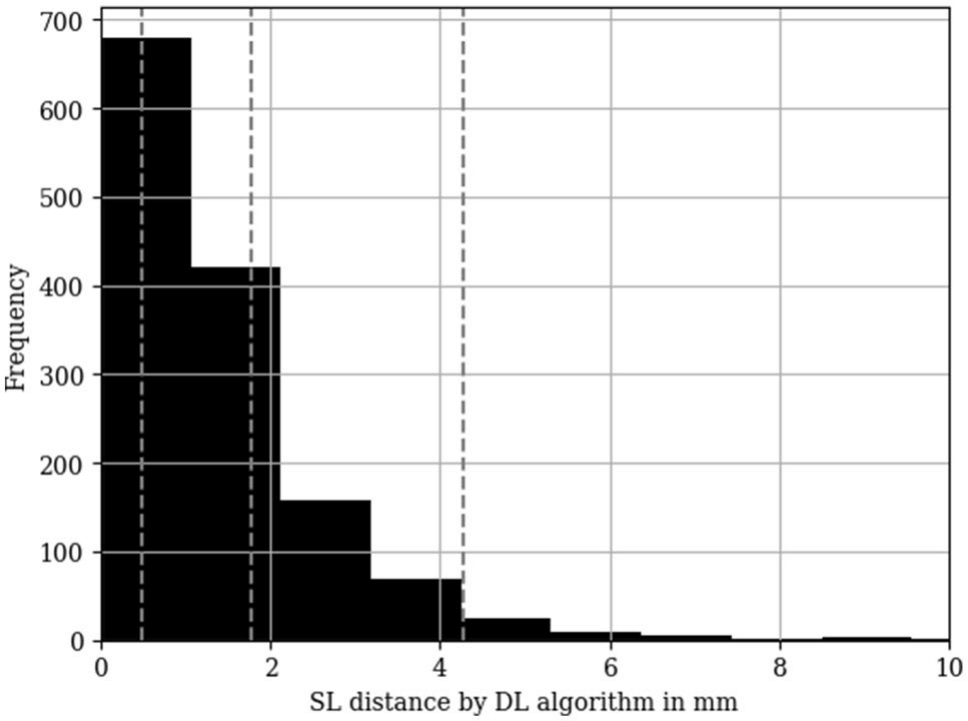
Histogram of SL distances measured by the DL algorithm in the study population (*n* = 364 patients; *n* = 1604 radiographs). Dashed lines mark the mean, the 5th and the 95th percentile. SL = scapholunate, DL = deep learning

**Fig. 4:**
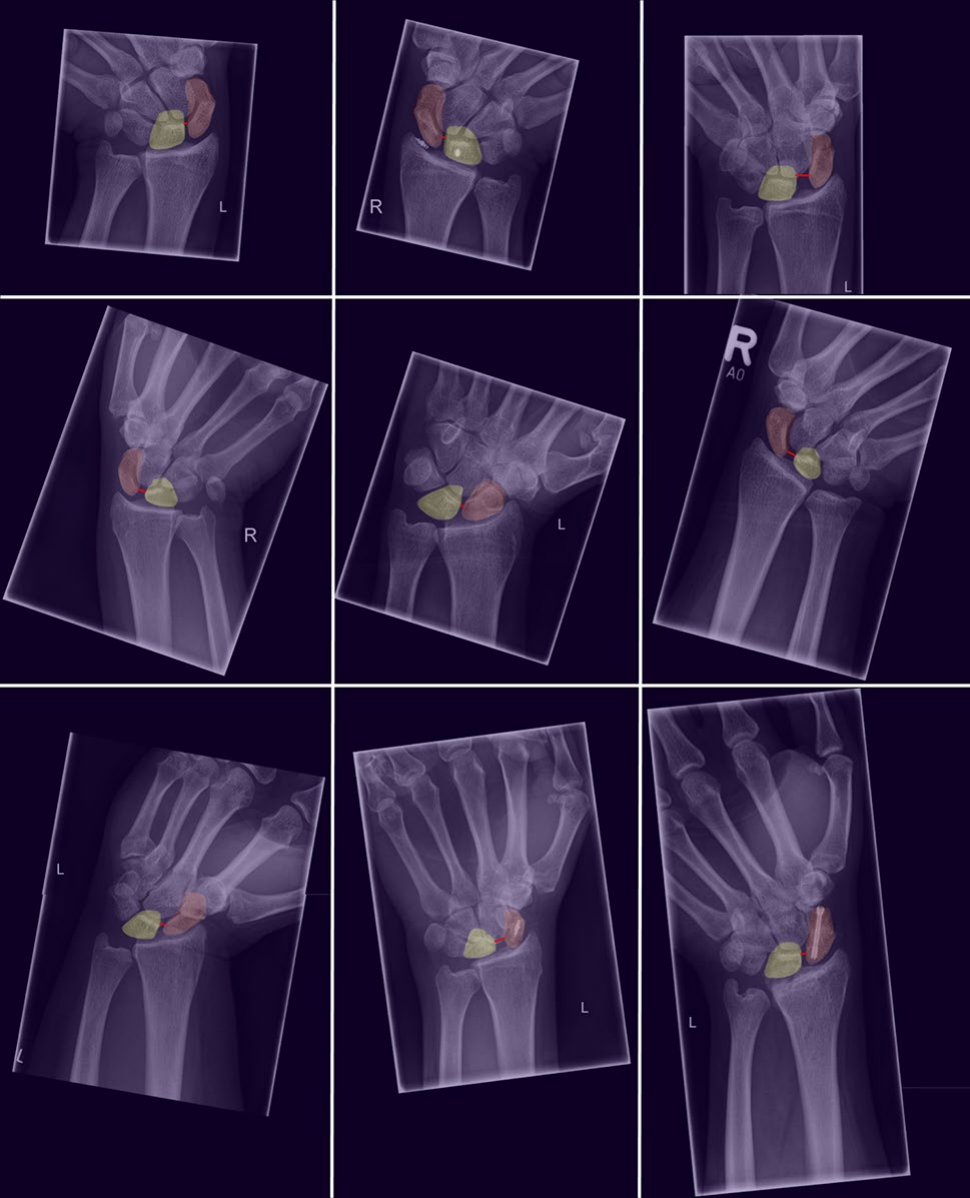
Radiographs of the wrist in dorsopalmar view with DL-based automated segmentation of the scaphoid and the lunate and automated measurement of the SL distance. Exemplarily shown is a spectrum of radiographs of different patients, right and left hands, different hand positioning, different tilting of the radiograph, different collimation, normal and widened SL distance and also radiographs with metal implants (Herbert screws and bone anchors). DL = deep learning, SL = scapholunate

There were no radiographs that could not be assessed by the DL algorithm (no output failure). The average SL distance measured by the DL algorithm was 2.3 mm, with a range of 0.0 mm to 15.9 mm. As shown in Fig. 3, the 5th and 95th percentiles were 0.2 mm and 4.0 mm, respectively. 22% of the reported measurements had a SL distance larger than 2 mm, and 11% had an SL distance greater than 3 mm (exemplary pictures are shown in Fig. 4).

Pearson's correlation coefficient between the measured SL distance by the DL algorithm and the human expert was 0.60 (*P* < 0.01). When removing measurements of the DL algorithm above 7 mm and 5 mm, the Pearson's correlation coefficient between the measured SL distance by the DL algorithm and the human expert was 0.80 (*P* < 0.01) and 0.81 (*P* < 0.01), respectively.

**Fig. 5:**
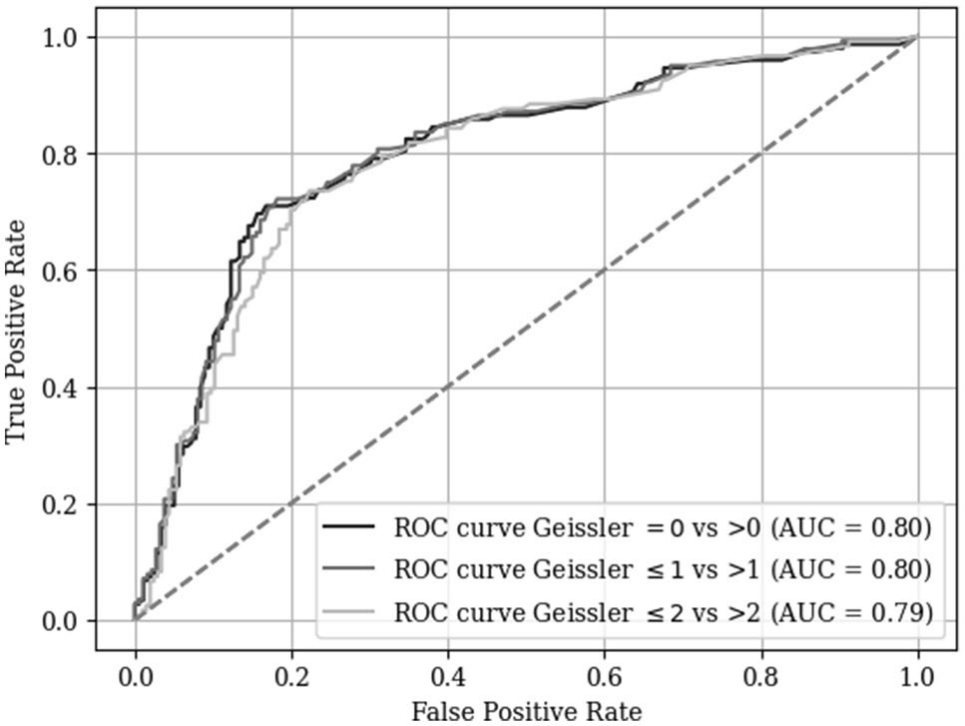
ROC curve of the DL algorithm for arthroscopically assessed SL integrity according to Geissler’s classification. ROC = receiver operating characteristics, AUC = area under the curve, DL = deep learning, SL = scapholunate ligament

The receiver operating characteristic (ROC) curve shows robust DL algorithm performance (see Fig. 5). The area under the ROC curve (AUC) is 0.80 for Geissler’s classification of 0 versus > 0 and ≤ 1 versus > 1, and 0.79 for ≤ 2 versus > 2.

The Spearman's order correlation coefficient between the DL measured SL distance and the Geissler’s classification was 0.52 (*P* < 0.01). Table 2 shows the sensitivity and specificity for predicting arthroscopic SL integrity based on SL distance data from the DL algorithm. When removing measurements of the DL algorithm above 7 mm (*n* = 9 radiographs excluded, which would correspond to an output failure of 0.56%), the sensitivity and specificity with optimal cut-off of ≤ 1.96 mm versus > 1.96 mm were 0.70 and 0.84 for Geissler’s classification of 0 versus > 0, 0.71 and 0.83 for ≤ 1 versus > 1, and 0.72 and 0.79 for ≤ 2 versus > 2. When removing predicted values above 5 mm (*n* = 24 radiographs excluded), the sensitivity and specificity were 0.67/0.86 (Geissler 0 vs > 0), 0.68/0.85 (Geissler ≤ 1 vs > 1), and 0.70/0.81 (Geissler ≤ 2 vs > 2).

**Table 2:** Sensitivity and specificity of the newly designed DL algorithm assessing the SL distance on dorso-palmar radiography as a surrogate parameter for the prediction of arthroscopic SL integrity

Geissler’s classification	Cut-off (mm)	Sensitivity	Specificity
0 versus > 0	≤ 1.96 versus > 1.96	0.71	0.83
0 versus > 0	≤ 2 versus > 2	0.70	0.84
0 versus > 0	≤ 3 versus > 3	0.45	0.91
≤ 1 versus > 1	≤ 1.96 versus > 1.96	0.72	0.82
≤ 1 versus > 1	≤ 2 versus > 2	0.71	0.82
≤ 1 versus > 1	≤ 3 versus > 3	0.46	0.90
≤ 2 versus > 2	≤ 1.96 versus > 1.96	0.74	0.78
≤ 2 versus > 2	≤ 2 versus > 2	0.72	0.78
≤ 2 versus > 2	≤ 3 versus > 3	0.47	0.87

Various cut-off values for the SL distance for the discrimination of different stages of Geissler’s classification [18] are reported

*DL* deep learning, *SL* scapholunate

## Discussion

SL tears are known as the most common cause of posttraumatic degenerative wrist arthritis [11]. As for the exclusion of osseous injuries, like fractures, emergency departments’ patients after an acute wrist trauma usually undergo plain radiography. If a SL tear is clinically suspected and depending on intra-individually different institutional standard operating procedures, static radiography may be supplemented by dynamic radiography, e.g. in radial/ulnar duction as in the underlying study. In the presented study, diagnostic accuracy of plain radiography was highest using a cut-off at 3 mm, e.g. a sensitivity of 77% and a specificity of 80% for the discrimination of SL tears Geissler’s stage ≤ 2 and > 2. This coincides with results in the literature reporting a sensitivity of 81% and a specificity of 80% [21] for the detection of SL dissociation in radiography.

Although the newly developed DL algorithm measured SL distance in a slightly different way—measuring the absolute shortest distance between scaphoid and lunate on each radiograph, whereas the experienced human reporter is assumed to have been able to detect overlap and thus measure the shortest distance between the cortical boundaries of the two bones—the newly developed DL algorithm correlated strongly positive with the human reporter (0.81; *P* < 0.01) and reached a comparable diagnostic accuracy, e.g. for the discrimination of Geissler’s stages ≤ 2 and > 2 the sensitivity was 74% and the specificity was 78%—without an output failure (radiographs for which the DL algorithm did not provide an adequate result). Hence, according to Geissler’s suggestions [19] the DL algorithm indicated in 83% correctly, if no therapy at all would be necessary, in 72%, if a at least a minimal invasive surgical therapy (‘Arthroscopic reduction and pinning’) would be necessary and in 74%, if an open surgical therapy (‘Open reduction and repair’) would be necessary. Remarkably, the results of the DL algorithm were unharmed by metal implants.

Of course, other non-invasive and invasive methods are clearly superior to radiography for the detection of SL tears: MRI is supposed to be the gold standard for the non-invasive diagnostics of a suspected SL tear with a wide range of reported diagnostic accuracy (sensitivity 45.7–75.7%; specificity 80.5–100.0%), thereby 3 T MRI is reported to be superior over 1.5 T MRI [22, 23]. These data of diagnostic accuracy for MRI seem low, an explanation could be that using MRI readers may claim to even detect slight alterations, that not necessarily need further management. Here it has to be mentioned that not all studies use the same definition, classification and stages of SL injury. Arthrography as an invasive diagnostical method is reported to be superior for the clarification of a suspected SL tear compared to MRI with a sensitivity of 82% and a specificity of 93% [22]. Arthroscopy as a surgical method remains the gold standard for SL diagnostics [14].

To the knowledge of the authors there are no similar studies on a DL algorithm for automated interpretation of the SL distance as a surrogate parameter for SL injury published so far. Studies on automated image interpretation in the field of MSK imaging had rather focused on fracture detection or bone age [2, 9, 24–28]. Development of AI algorithms for automated assessment of the alignment between different bones are rare: Some authors claim to reach a reliable radiological assessment of an AI-based software comparing to human raters on automated assessment of hip dysplasia [29]. Thereby 6 out of 136 patients (4.4%) could not be assessed by the AI algorithm due to technical reasons, although among others, patients after prior surgical intervention, presumably meaning with metal implants, and patients with avascular necrosis had been excluded. In contrast, other groups aiming to automate the assessment of measurements on long leg radiographs as for example the hip-knee-ankle angle concluded after a first study on patients without metal implants [7], that reliable assessment in patients with total knee arthroplasties would be possible, although on the other hand in that study patients with unicondylar knee arthroplasty, “incorrect positioning” and “poor visibility” were excluded, and again the AI software produced an output failure of 4% [30].

As a limitation of the reported results, it has to be mentioned that like in other studies the newly developed DL algorithm for the automated assessment of the SL integrity is not error-free. Nevertheless, the authors believe that algorithms like this may become an important tool for medics as they might suggest an image interpretation and medics will keep the authority to accept or overrule this suggestion. Also, an algorithm may direct the attention of unexperienced medics to relevant pathologies and support or even improve their image interpretation, while experienced readers may save time not having to measure simple distances or angles. Further limitations are the retrospective study design and the relatively small data-set of *n* = 201, that has been used for fine-tuning of the DL algorithm compared to a number of mostly several thousand that have been used in others of the cited studies. Nevertheless, the algorithm worked surprisingly robust, unharmed by for example metal implants. It should also be mentioned that the relatively small number of evaluation cases (*n* = 364 patients) may limit the generalizability of the findings to other populations. However, it is worth noting that the evaluation data set included a large number of radiographs (*n* = 1604), including radiographs from external institutions, which strengthens the robustness of the DL algorithm. Of course, the algorithm may be further improved due to more training and a prospective evaluation of the algorithm in clinical routine could strengthen the results. Finally, only a single DL algorithm was evaluated in this study. The field of DL is rapidly evolving, and new, better-performing models are constantly being developed that could potentially further improve the reported results. Therefore, it is possible that newer DL models may outperform the algorithm used in this study.

In regard of the potential and the common use of radiography after an acute wrist trauma aiming to exclude fractures and to deliver an orientation as for triaging patients for further diagnostics, e. g. MRI clarification of suspected SL tears, the newly developed DL algorithm showed high diagnostic accuracy for the assessment of SL integrity compared to arthroscopic correlation and it might become a supporting tool for interpreting wrist surgeons and radiologists.

## Abbreviations

AI: Artificial intelligence
AP: Average precision
AUC: Area under the ROC curve
COCO: Common objects in context
DL: Deep learning
MRI: Magnetic resonance imaging
MSK: Musculoskeletal
PACS: Picture archiving and communication system
ROC: Receiver operating characteristic
SL: Scapholunate ligament
SLAC: Scapholunate advanced collapse
